# The efficacy of corticosteroids therapy in patients with moderate to severe SARS-CoV-2 infection: a multicenter, randomized, open-label trial

**DOI:** 10.1186/s12931-021-01833-6

**Published:** 2021-09-15

**Authors:** Mostafa Ghanei, Masoud Solaymani-Dodaran, Ali Qazvini, Amir Hosein Ghazale, Seyed Amin Setarehdan, Seyed Hassan Saadat, Hassan Ghobadi, Saeed Hoseininia, Maryam Elahikhah, Ali Hossein Samadi, Yousef Imani, Ensieh Vahedi, Farhang Babamahmoodi, Fatemeh Tajik Rostami, Mohammad Hossein Azimzadeh Ardebili, Akram Ansarifar, Fatemeh Fallahpoor Golmaei, Atieh Asadollah

**Affiliations:** 1 Chemical Injuries Center, Systems Biology and Poisoning Institute, Baqiyatallah University of Medical Sciences, MollaSadra St., Tehran, Iran; 2 Minimally Invasive Surgery Research Center, Hazrat-e-Rasool Hospital, Iran University of Medical Science, Tehran, Iran; 3 Division of Epidemiology and Public Health, University of Nottingham, Nottingham, NG7 2UH UK; 4 School of Public Health, Iran University of Medical Science, Tehran, Iran; 5 Trauma Research Center, Baqiyatallah University of Medical Sciences, Tehran, Iran; 6 Student Research Committee, Baqiyatallah University of Medical Sciences, Tehran, Iran; 7 Behavioral Sciences Research Center, Life Style Institute, Baqiyatallah University of Medical Sciences, Tehran, Iran; 8 Department of Internal Medicine (Pulmonary Division), Faculty of Medicine, Ardabil University of Medical Sciences, Ardabil, Iran; 9 Department of Internal Medicine, Faculty of Medicine, Ardabil University of Medical Sciences, Ardabil, Iran; 10 Chemical Injuries Center, Systems Biology and Poisoning Institute, Baqiyatallah University of Medical Sciences, Tehran, Iran; 11 Antimicrobial Resistance Research Center, Communicable Diseases Institute, Mazandaran University of Medical Sciences, Sari, Iran; 12 Master of Clinical Psychology, Mazandaran, Iran; 13 Firoozabadi Clinical Research Development Unit (FCRDU), Iran University of Medical Sciences, Tehran, Iran

**Keywords:** COVID-19, Corticosteroids, Low dose prednisolone, Anti-inflammatory drugs

## Abstract

**Background:**

We performed a multicenter, randomized open-label trial in patients with moderate to severe Covid-19 treated with a range of possible treatment regimens. Methods: Patients were randomly assigned to one of three regimen groups at a ratio of 1:1:1. The primary outcome of this study was admission to the intensive care unit. Secondary outcomes were intubation, in-hospital mortality, time to clinical recovery, and length of hospital stay (LOS). Between April 13 and August 9, 2020, a total of 336 patients were randomly assigned to receive one of the 3 treatment regimens including group I (hydroxychloroquine stat, prednisolone, azithromycin and naproxen; 120 patients), group II (hydroxychloroquine stat, azithromycin and naproxen; 116 patients), and group III (hydroxychloroquine and lopinavir/ritonavir (116 patients). The mean LOS in patients receiving prednisolone was 5.5 in the modified intention-to-treat (mITT) population and 4.4 days in the per-protocol (PP) population compared with 6.4 days (mITT population) and 5.8 days (PP population) in patients treated with Lopinavir/Ritonavir.

**Results:**

The mean LOS was significantly lower in the mITT and PP populations who received prednisolone compared with populations treated with Lopinavir/Ritonavir (p = 0.028; p = 0.0007). We observed no significant differences in the number of deaths, ICU admission, and need for mechanical ventilation between the Modified ITT and per-protocol populations treated with prednisolone and Lopinavir/Ritonavir, although these outcomes were better in the arm treated with prednisolone. The time to clinical recovery was similar in the modified ITT and per-protocol populations treated with prednisolone, lopinavir/ritonavir, and azithromycin (P = 0.335; P = 0.055; p = 0.291; p = 0.098).

**Conclusion:**

The results of the present study show that therapeutic regimen (regimen I) with low dose prednisolone was superior to other regimens in shortening the length of hospital stay in patients with moderate to severe COVID-19. The steroid sparing effect may be utilized to increase the effectiveness of corticosteroids in the management of diabetic patients by decreasing the dosage.

## Background

Coronavirus disease-19 (COVID-19) has resulted in a global pandemic. The disease is associated with a series of clinical settings from asymptomatic infections to mild and severe manifestations, contributing to a significant morbidity and mortality, as well as strain on intensive care unit (ICU) capacity [1–3].

As the pandemic progresses, overwhelming body of evidence regarding the pathological pattern of the disease and the potential impact of immunomodulatory strategies [4] has suggested that cytokine storm may be the main cause of disease progression (the severity and clinical outcomes) leading to lung injury and organ failure [5].

Supportive and adjuvant therapy have been recommended for the treatment of COVID-19 due to the absence of specific treatments such as antivirals. The histological pattern of pulmonary edema, hyaline membrane formation, and acute fibrinous and organizing pneumonia (AFOP) which characterize acute lung injury of the disease [6–8] suggest that timely and appropriate use of corticosteroid may be beneficial in patients with severe disease.

The severity and pathophysiology of the disease have been linked to hyperinflammation, therefore, a combination therapy of off-label drugs such as immunosuppressor/immunomodulator*,* inflammatory cytokines antagonists, and nonsteroidal anti-inflammatory drugs (NSAIDs) may be of utmost importance in mitigating the potential effects of cytokine storm in the inflammation-driven damaging phases of COVID-19.

Corticosteroids suppress inflammation-induced lung injury by inhibiting lung inflammation in critically ill patients [9–11]. On February 6th, 2020, researchers from Edinburgh published a short review of clinical data regarding the outcomes of corticosteroid therapy in respiratory diseases [12]. They concluded that there is no clinical data that supports the use of corticosteroids in COVID-19 patients. This was recapitulated by WHO who discouraged the use of steroids in Covid-19. Rapidly, the response from physicians from Wuhan and Beijing appeared. They opposed the liberal use of corticosteroids and recommend short courses of corticosteroids at low-to-moderate dose, used prudently, for the most severely ill patients [13]. The most robust evidence supporting the use of corticosteroids therapy came from the COVID-19 RECOVERY Trial. In this trial, the 28-day mortality rate for COVID-19 patients requiring either invasive mechanical ventilation or oxygen therapy was decreased in patients treated with low dose dexamethasone (6 mg once daily) [14]. This finding led to the recommendation of corticosteroids therapy for the treatment of COVID-19 patients by the WHO (WHO, 2020).

Short term administration of low-to-moderate dose of corticosteroids has been recommended for critically ill COVID-19 patients by a Chinese expert panel based on their experience [13]. Short course corticosteroid therapy is relatively safe, despite a potential secondary hyperglycemia, while its long-term use may be linked to glaucoma, hypertension, cataracts, increased risk of infection, and fluid retention [15].

Over the past months of the pandemic, improved clinical outcomes were observed by the use of early short courses of methylprednisolone, especially at low dose (0.5–2 μg/kg/day) for COVID-19 patients in previous studies [16, 17]. However, in most of these studies, there were lack of follow up data or the cohort size was small, and thus the use of short courses of methylprednisolone needs further evaluation in randomized clinical trial. There still remain uncertainties regarding the use of corticosteroids, such as potential risks of corticosteroid administration, particularly in diabetic patients, as well as the association with arterial hypertension [18]. In diabetic patients, the administration of corticosteroids should be taken into consideration because it could worsen glycemic control and insulin resistance, and induce hyperglycemia [19]. Therefore, short courses of low dose methylprednisolone may provide better outcome in diabetic patients.

The evidence provided by the RECOVERY trial recommended that RCTs be performed based on the PICO criteria (e.g., age stratification, oxygen therapy status and adverse effects, etc.).

Furthermore, the efficacy of corticosteroid therapy probably depends on the dosing, timing of corticosteroids initiation, and the duration of use in the right patient.

Therefore, in this multicenter, randomized, open-label trial, we investigated whether short course administration of low-dose prednisolone would be beneficial in improving clinical outcomes of patients with SARS-CoV-2 infection. We also determined the adverse events associated with the prednisolone outcome.

## Material and methods

### Trial design and oversight

This study was a multicenter, randomized, open-label, three-arm trial performed in 6 centers from April 13 to August 9, 2020, to evaluate the effects of potential therapeutic regimens in hospitalized patients with COVID-19 in a 1:1:1 ratio to receive either treatment and control.

Hospitalized patients, 16 years of age or older, were enrolled in the trial if they had a positive polymerase-chain-reaction (PCR) assay and oxygen saturation (Spo_2_) less than 94% or less.

Patients were excluded if they had a history of receiving any medications (i.e., Immunosuppressive drugs, systemic steroids, chemotherapy drugs, Hydroxychloroquine, Lopinavir/Ritonavir, Ribavirin, and Oseltamivir) for COVID-19 in the last month. Patients with uncontrolled diabetes or asthma were excluded from this trial. Patients with other comorbidities were not excluded from the study if they had received the drug for their condition. Other exclusion criteria included chronic renal or liver disease, gastrointestinal hemorrhage, untreated bacterial infection, pregnancy or breast-feeding, and QT interval ≥ 500 ms. COVID-19 patients who were ill with less than 48 h were also excluded.

### Randomization and treatment

Using a stratified block randomization method with variable block sizes of 6 and 9, we randomly assigned patients in a 1:1:1 ratio to one of three treatment regimens. A 4-digits unique code was assigned to each eligible patient by a central allocation mechanism for subject identification on CRF forms. We applied sealed envelopes to protect the randomization sequence.

In the first group (regimen I), patients received 400 mg hydroxychloroquine stat and then the following medications were administered daily for 5 days including prednisolone (25 mg prednisolone daily), 250 mg azithromycin (two tablets on the first day and then 250 mg daily), and 250 mg naproxen (twice a day). In this group, Prednisolone was gradually tapered to 5 mg per week after discharge for reduction of readmission based on the decision of the DSMB members because the patients met the discharge criteria, but pulmonary involvement maybe remained.

In group 2 (regimen II), patients received 400 mg hydroxychloroquine stat, in addition to 250 mg azithromycin (two tablets on the first day and then 250 mg daily), and 250 mg naproxen (twice a day) for 5 days. Patients in regimen I and II groups also received 40 mg of pantoprazole tablets or capsules daily during treatment to prevent gastrointestinal complications. The treatment protocols in these groups were continued for 10 days based on patients' response to treatment (criteria for discharge) if needed. Thus, this was the description of what were the criteria for extending treatment time.

In the third group (regimen III), patients received 400 mg hydroxychloroquine stat plus 200/50 lopinavir/ritonavir twice a day for 7 days. This treatment protocol was continued for 14 days if needed. It is noteworthy that we assigned patients to hydroxychloroquine as standard of care in Iran, when there was no evidence against its beneficial effects at the time of the study.

### Data management

The monitoring of the participating centers was done through on-site monitoring and remote monitoring using special software. National professional bodies and the chief investigator played a key role in coordinating between the participating centers. All data were recorded daily from all centers using the electronic CRF form designed using special software (local client software). The data were then transferred to a central database as the latest version of data. The electronic CRF form had the same structure and design as paper CRFs. An application was also created to display all the contents of the CRF forms in the central database by which discrepancies and errors were identified and reported to the clinical unit for correction (remote monitoring). All patients were also monitored at least once daily by trained physicians. All adverse drug reactions were recorded and reviewed by the Safety and Data Monitoring Committee.

### Outcome measures

The number of admissions to intensive care unit was considered as the primary outcome. Patients with the following clinical conditions were transferred to the intensive care unit for advanced treatment and support: 1, Decreased consciousness (Glasgow Coma Scale [GCS] less than 12); 2, Shock (systolic blood pressure less than 90 and diastolic blood pressure less than 60); 3, Hypoxia (O_2_saturation less than 90%) and unresponsive to non-rebreather mask. Decisions to intubation were made by the attending physician. Secondary outcomes included the length of hospital stay (LOS), death during admission, intubation in ICU, and time to clinical recovery. Clinical recovery was defined as being medically stable and ready for discharge from the hospital, determined by the attending physician. Patients with oxygen saturation > 93% or not requiring oxygen therapy were considered clinically stable. Safety outcomes were adverse events, serious adverse events, and premature discontinuation from the trial.

### Safety and efficacy population

**Fig. 1:**
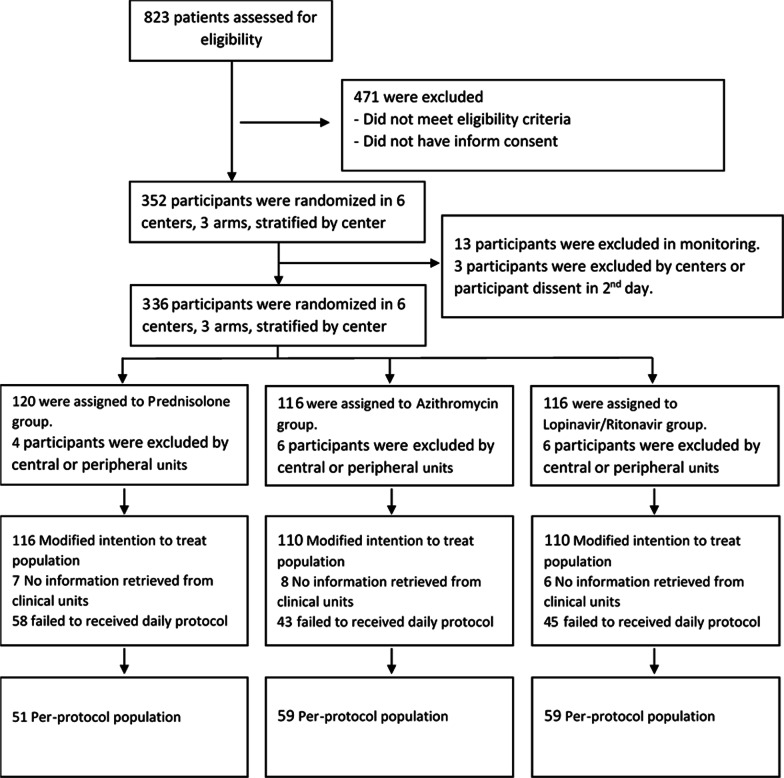
Participants flow diagram

Sixteen patients from two participating centers were excluded from the study during the central team visit due to non-compliance with the inclusion criteria and hence were not included in the modified ITT population. Patients were excluded from the population and recorded in the relevant CRF if their treatment process was deviated from the protocol (Fig. 1). Therefore, our pre-protocol population includes all those who were completely treated according to the protocol. Patients who received additional treatment, including pulsed or intravenous steroid therapy were excluded from the pre-protocol population (Table 5).

The per-protocol population was obtained after the exclusion of the following patients: a) 149 patients were excluded from the protocol due to prescription of treatment in addition to the study protocol. Patients who have taken different doses of corticosteroids were excluded from the study. b) Patients whose information was not accessible for any reason were excluded from the ITT population (21 patients), resulting in the formation of the Modified ITT (Fig. 1).

Safety comparisons were performed in the Modified ITT population and efficacy outcomes were compared in both the Modified ITT and per-protocol populations.

### Statistical analysis

The study was stopped according to the decision of the scientific committee in the Data and Safety Monitoring Board (DSMB) meeting, before reaching a sufficient sample size because the use of corticosteroids even at higher doses than this study was added to the protocol of Iran at that time for patients with COVID-19 disease and other studies mentioned the usefulness of corticosteroids for the treatment of hospitalized patients with COVID-19. Therefore, continuing the study based on randomization in both arms not only deprived patients from corticosteroids but also was not morally right. Due to changes in COVID-19 treatment protocols, adherence to the protocol was clinically impossible. The intention-to-treat principle was applied for all analysis of patients randomly assigned to each group.

To ensure that participants have been randomly assigned to groups, all variables were compared between the ITT and Modified ITT populations (baseline population) of the three groups before the initiation of the intervention. Based on the defined populations, a participant flow diagram was plotted. Outcome analysis was performed for both modified ITT and per-protocol populations by two-by-two comparison of the groups. In addition to the analysis of the proportions of death, ICU admission and intubation, by multiple comparisons and two-by-two comparison of the groups (chi squared test), these outcomes were also analyzed using the survival analysis approach.

Death and initiation of mechanical ventilation outcomes were investigated by using the Kaplan–Meier estimates and the time to discharge by Nelson-Aalen. For both defined outcomes, the log-rank statistic was used (i.e., the log-rank test). We conducted a stratified log-rank test of time to recovery for the prednisolone group versus Lopinavir/Ritonavir group.

Estimates of hazard ratios and their 95% confidence intervals were done by using Cox proportional hazard model. Proportional hazard assumption was performed for each model using three methods; proportional hazard, Log minus log survival plots, and proportional hazard (PH) test if the number of outcomes was sufficient in three groups. Cox proportional hazard analysis was used to compare the time to occurrence of events. Cox proportional hazard analysis is a multivariate model-based analysis and the relation of the main variables were adjusted by other confounders.

Linear regression of General Estimation Equation (GEE) and logistic regression were used to assess the change in total symptoms and the need for oxygen support during hospitalization, respectively. Due to the fact that the investigators of this study were interested in finding, superiority of treatment regimen, the percentage of need for intensive care was compared among the groups and a statistically significant difference was reported.

Regarding differences in the prognosis of patients, subgroup analysis was performed depending on the presence of comorbidity, age, and sex. After supplying 30% of the samples, Interim analysis was performed and DSMB members reviewed analyses of the study data. Steering committee concluded that enrollment of patients to the groups be continued after correction of alpha error based on statistical considerations. Statistical analyses were done using Stata version 11 (STATA Corporation).

## Results

### Patients

We recruited 352 eligible SARS-CoV-2 patients who were randomly assigned to the Steroid + azithromycin (120 patients), azithromycin (116), and lopinavir/ritonavir (116) groups in 6 centers (Table 1 and Fig. 1). Sixteen patients from two participating centers were excluded from the study during the central team monitoring due to non-compliance with the inclusion criteria. Therefore, a total of 336 patients were randomly assigned to receive 3 different treatment regimens. Of these eligible patients, 116 were assigned to the Prednisolone group (Regimen I), 110 were assigned to the azithromycin group (Regimen II), and 110 to lopinavir/ritonavir groups (Regimen III) as modified intention to treat population. Moreover, 169 participants were considered for complete 5-day therapy with the 3 regimens as per-protocol population.

**Table 1:** Participants of study

Assigned intervention	No. randomized patients	No. of lost patients	Modified ITT population	No. of subjects deviating from the protocol	Per-protocol population
Steroid + Azithromycin	120	4	116	52	64
Azithromycin	116	6	110	59	51
Lopinavir/ Ritonavir	116	6	110	59	51
Total	352	16	336	170	166

The baseline demographic and disease history of the ITT populations are summarized in Table 2. Table 3 shows the clinical characteristics of the Modified ITT population at the time of admission and during hospitalization. Baseline laboratory results of the Modified ITT population of the three intervention groups have provided in Table 4.

**Table 2:** Demographic information, disease history and vital signs in the baseline in three different groups in the ITT population

	Steroid + Azithromycin	Azithromycin	Lopinavir/Ritonavir	Total	P-value
Age—mean (SD)*	58.2 (17.2)	57.6 (15.6)	58.4 (16.0)	58.1 (16.3)	0.889
Sex (Male)—no. (%)	57 (49.1)	55 (50.0)	61 (55.5)	173 (51.5)	0.307
BMI—mean (SD)	28.8 (4.7)	29.4 (5.6)	27.7 (5.8)	28.6 (5.4)	0.068
Smoking—no. (%)					
Never-smoker	38 (82.6)	36 (70.6)	32 (72.7)	106 (75.2)	0.625
Current smoker	2 (4.4)	6 (11.8)	5 (11.4)	13 (9.2)	
Ex-smoker	6 (13.0)	9 (17.7)	7 (15.9)	22 (15.6)	
Total	46 (100.0)	51 (100.0)	44 (100.0)	141 (100.0)	
Comorbidities—no. (%)					
Hypertension	23 (19.8)	30 (27.3)	30 (27.3)	83 (24.7)	0.638
Diabetes	13 (11.2)	14 (12.7)	14 (12.7)	41 (12.2)	0.964
Chronic heart disease	7 (6.0)	15 (13.6)	8 (7.3)	30 (8.9)	0.122
Chronic lung disease, not asthma	4 (3.5)	6 (5.5)	5 (4.6)	15 (4.5)	0.824
Chronic Kidny Disease	1 (0.9)	1 (0.9)	2 (1.8)	4 (1.2)	0.794
Mild liver disease	2 (1.7)	1 (0.9)	1 (0.9)	4 (1.2)	0.125
Rheumatologic disease	0 (0.0)	1 (0.9)	0 (0.0)	1 (0.3)	0.367
Chronic neurologic disease	5 (4.3)	2 (1.8)	4 (3.6)	11 (3.3)	0.502
Vital signs on admission—no. (%)					
Fever °C—mean (SD)	37.15 (0.69)	37.20 ((0.71)	37.17 (0.69)	37.18 (0.70)	0.884
Heart rate—mean (SD)	87.45 (14.96)	89.61 (15.23)	87.33 (14.48)	88.11 (14.87)	0.491
Respiratory rate—median (IQR)	18 (18–20)	19 (18–22)	19 (18–22)	18 (18–21)	0.704
O_2_ Saturation %—median (IQR)	90 (88–92)	90 (87–92)	90 (87–92)	90 (88–92)	0.617
Systolic BP mmHg—mean (SD)	121.84 (16.8)	124.14 (15.4)	123.78 (15.9)	122.91 (16.0)	0.594
Diastolic BP mmHg—mean (SD)	76.6 (10.1)	77.97 (9.9)	76.32 (9.7)	76.95 (9.9)	0.411

**Table 3:** Comparison of patients' symptoms at the time of admission and during hospitalization in the Modified ITT population

Symptoms—no. (%)	Group				P-value
	Steroid + Azithromycin	Azithromycin	Lopinavir/Ritonavir	Total	
Respiratory distress	17 (17.9)	13 (13.5)	6 (6.0)	36 (12.4)	0.041
Chill	33 (34.7)	36 (37.5)	35 (35.0)	104 (35.7)	0.910
Cough	65 (68.4)	70 (72.9)	78 (78.0)	213 (73.2)	0.341
Dyspnea	63 (66.3)	66 (68.8)	66 (66.0)	195 (67.0)	0.866
Chest pain	23 (24.2)	30 (31.3)	29 (29.0)	82 (28.2)	0.671
Anorexia	59 (62.1)	62 (64.6)	61 (61.0)	182 (62.5)	0.817
Diarrhea	17 (17.9)	21 (21.9)	21 (21.0)	59 (20.3)	0.779
Vomiting	9 (9.5)	17 (17.7)	13 (13.0)	39 (13.4)	0.261
Abdominal pain	15 (15.8)	15 (15.6)	16 (16.0)	46 (15.8)	0.991
Sore throat	12 (12.6)	13 (13.5)	14 (14.0)	39 (13.4)	0.853
Myalgia	45 (47.4)	48 (50.0)	56 (56.0)	149 (51.2)	0.499
Arthralgia	8 (8.4)	21 (21.9)	12 (12.0)	41 (14.1)	0.025
Fatigue	54 (56.8)	54 (56.3)	62 (62.0)	170 (58.4)	0.671
Headache	32 (33.7)	32 (33.3)	38 (38.0)	102 (35.1)	0.745

**Table 4:** Laboratory evaluation at the baseline in the Modified ITT population

	Steroid + Azithromycin		Azithromycin		Lopinavir/Ritonavir		Total		P-value
	N	Mean (SD)	N	Mean (SD)	N	Mean (SD)	N	Mean (SD)	
Blood cell count									
WBC	87	6.74 (3.28)	91	6.53 (2.76)	93	6.69 (3.35)	271	6.65 (3.13)	0.253
Lymphocyte	84	1.36 (0.86)	89	1.32 (0.89)	89	1.61 (2.15)	262	1.43 (1.44)	0.919
Neutrophyl	73	4.66 (2.67)	80	4.76 (2.47)	83	5.46 (8.16)	236	4.98 (5.25)	0.117
Eosinophyl	35	0.16 (0.13)	43	0.21 (0.56)	47	0.22 (0.58)	125	0.2 (0.49)	0.580
Basophyl	3	0.31 (0.28)	4	0.02 (0.01)	3	0.14 (0.21)	10	0.14 (0.21)	0.368
Platelet	77	204.25 (72.36)	87	190.4 (75.86)	82	198.88 (72.76)	246	197.56 (73.67)	0.658
Hb	84	13.32 (2.27)	89	13.56 (2.2)	93	13.64 (1.72)	266	13.51 (2.07)	0.282
HCT	82	40.36 (5.26)	87	40.57 (5.59)	88	40.35 (4.97)	257	40.43 (5.26)	0.635
Liver function									
ALT	70	43.36 (27.24)	75	51.94 (43.97)	67	54.78 (57.48)	212	50 (44.47)	0.398
AST	70	41.07 (33.75)	75	44.35 (31.64)	67	45.22 (36.17)	212	43.54 (33.7)	0.603
ALKPH	63	191 (71.31)	66	208.3 (93.59)	57	184.19 (64.78)	186	195.05 (78.46)	0.067
BILI (Dir)	33	0.29 (0.15)	38	0.39 (0.63)	27	0.26 (0.12)	98	0.32 (0.41)	0.108
BIL (Total)	33	0.8 (0.49)	40	45.04 (262.34)	27	0.68 (0.32)	100	18.47 (166.1)	0.319
Other laboratory parameters									
CRP	62	27.78 (49.67)	66	37.16 (73.32)	68	31.11 (62.07)	196	32.09 (62.47)	0.649
ESR	51	47.78 (26.34)	63	37.98 (26.33)	57	40.35 (23.41)	171	41.7 (25.58)	0.816
FERRITINE	37	530.78 (728.58)	46	720.67 (603.16)	43	426.12 (380.65)	126	564.39 (590.28)	0.050
LDH	74	641.51 (394.26)	69	628.81 (237.53)	70	651.93 (407.81)	213	640.82 (354.74)	0.768
PT	63	13.41 (2.66)	70	13.99 (2.85)	66	13.31 (2.26)	199	13.58 (2.61)	0.278
PTT	63	34.84 (8.56)	68	32.72 (5.75)	66	35.89 (11.38)	197	34.46 (8.9)	0.941
INR	61	1.13 (0.23)	68	1.12 (0.15)	61	1.07 (0.14)	190	1.11 (0.18)	0.233
BUN	81	17.2 (10.44)	89	16.88 (8.96)	80	16.74 (8.9)	250	16.94 (9.41)	0.328
CR	82	1.08 (0.73)	91	1.01 (0.29)	84	2.18 (10.92)	257	1.41 (6.25)	0.471
Uric acid	21	6.77 (6.52)	24	6.03 (4.24)	21	33.15 (109.88)	66	14.9 (62.39)	0.344
BS	69	130.33 (52.07)	64	130.63 (51.33)	63	128.65 (63.84)	196	129.89 (55.61)	0.351
HBA1C	12	6.93 (1.84)	13	7.75 (1.97)	15	6.95 (1.84)	40	7.2 (1.87)	0.868
Na	80	137.99 (4.57)	81	138.21 (4.28)	84	138.12 (4.91)	245	138.11 (4.58)	0.990
K	79	4.03 (0.47)	84	10.63 (47.23)	84	3.99 (0.53)	247	6.26 (27.61)	0.177
Ca	35	8.81 (0.73)	39	8.91 (0.79)	43	8.74 (0.64)	117	8.82 (0.72)	0.677
Mg	33	2.02 (0.27)	36	5.28 (19.16)	38	2.38 (1.99)	107	3.24 (11.17)	0.391
Phosphorus	31	3.45 (0.91)	34	3.44 (0.64)	36	3.44 (0.6)	101	3.44 (0.72)	0.879
Vitamine D	12	60.08 (63.62)	15	127.27 (191.15)	14	90.64 (97.16)	41	95.1 (133.16)	0.370
TROPONINE	44	27.23 (153.31)	48	4.84 (29.35)	55	0.24 (1.03)	147	9.82 (85.64)	0.319
DDIMER	36	202.23 (427.91)	34	133.02 (322.12)	33	122.08 (329.15)	103	153.71 (362.88)	0.504

In addition to the drugs described in our treatment protocol (regimen), additional drugs were prescribed to the patients in different centers due to the unavailability of specific treatment (Table 5), therefore, what was emphasized in this study was that these additional treatments should be indicated on the same condition in all three groups. Due to the open labeling of the study, other treatments used in this trial may be affected by the type of intervention, so they are not included in the baseline comparison.

**Table 5:** List of additional drug modalities in the modified ITT population

Other Treatments received during study n (%)	Steroid + Azithromycin (N = 116)	Azithromycin (N = 110)	Lopinavir/Ritonavir (N = 110)	Total (N = 336)	P-value
Oral steroid	6 (5.17)	11 (10.0)	16 (14.6)	33 (9.8)	0.118
Intravenous steroid	8 (6.9)	15 (13.6)	6 (5.5)	29 (8.6)	0.032
Steroid pulse therapy	12 (10.3)	10 (9.1)	3 (2.7)	25 (7.4)	0.055
Interferone	1 (0.9)	1 (0.9)	0 (0)	2 (0.6)	0.578
NSAID	8 (6.9)	10 (9.1)	20 (18.2)	38 (11.3)	0.038
Anticoagolant	61 (52.6)	63 (57.3)	69 (62.7)	193 (57.4)	0.881
Bronchodilator	21 (18.1)	24 (21.8)	30 (27.3)	75 (22.3)	0.287
Plasmapheresis	4 (3.5)	1 (0.9)	3 (2.7)	8 (2.4)	0.386
Favipiravir	1 (0.9)	0 (0)	1 (0.9)	2 (0.6)	0.610
Hyperimmune plasma	4 (3.5)	0 (0)	2 (1.8)	6 (1.8)	0.141
CinnoRA	7 (6.0)	6 (5.5)	4 (3.6)	17 (5.1)	0.547
Hemoperfiosion	0 (0)	1 (0.9)	2 (1.8)	3 (0.9)	0.354
Actemra	0 (0)	1 (0.9)	3 (2.7)	4 (1.2)	0.163

In this study, that patients in the Lopinavir/Ritonavir group (III) and the azithromycin group (II) received more oral or intravenous steroids and anticoagulants than the prednisolone group (Regimen I), while pulse steroid therapy was more prescribed in the prednisolone group (Table 5). Although there does not appear to be a significant difference between the study groups in terms of additional medication us, this slight difference may be due to the compensatory measures of the treatment team in the regimen I or regimen II.

Overall, death occurred in 16 patients in the modified ITT population;4 patients ingroup I, 6 patients in group II, and6 patients in group III (Chi2 = 0.67; df = 1; P = 0.71), (Table 6). Eighteen patients were admitted to the intensive care unit. Of these, 6 underwent intubation (group I = 2 cases, group II = 1, and group III = 3). There was no statistically significant difference between the three groups in terms of need for intubation (Table 6).

**Table 6:** Efficacy outcomes in the Modified ITT and protocol populations

Outcomes in modified ITT	Steroid + Azithromycin (N = 116)	Azithromycin (N = 110)	Lopinavir/Ritonavir (N = 110)	Total (N = 336)	P value
Death	4	6	6	16	Chi2 = 0.67; df = 1; P = 0.71
Admission to ICU	5	6	7	18	Chi2 = 0.47; df = 1; P = 0.79
Intubation declared	2	1	3	6	Chi2 = 1.14; df = 1; P = 0.57

Outcomes in per-protocol	Steroid + Azithromycin (N = 64)	Azithromycin (N = 51)	Lopinavir/Ritonavir (N = 51)	Total (N = 166)	P value
Death	2	1	3	6	Chi2 = 1.20; df = 1; P = 0.55
Admission to ICU	1	3	4	8	Chi2 = 2.62; df = 1; P = 0.27
Intubation declared	0	1	2	3	Chi2 = 0.89; df = 1; P = 0.64

**Fig. 2:**
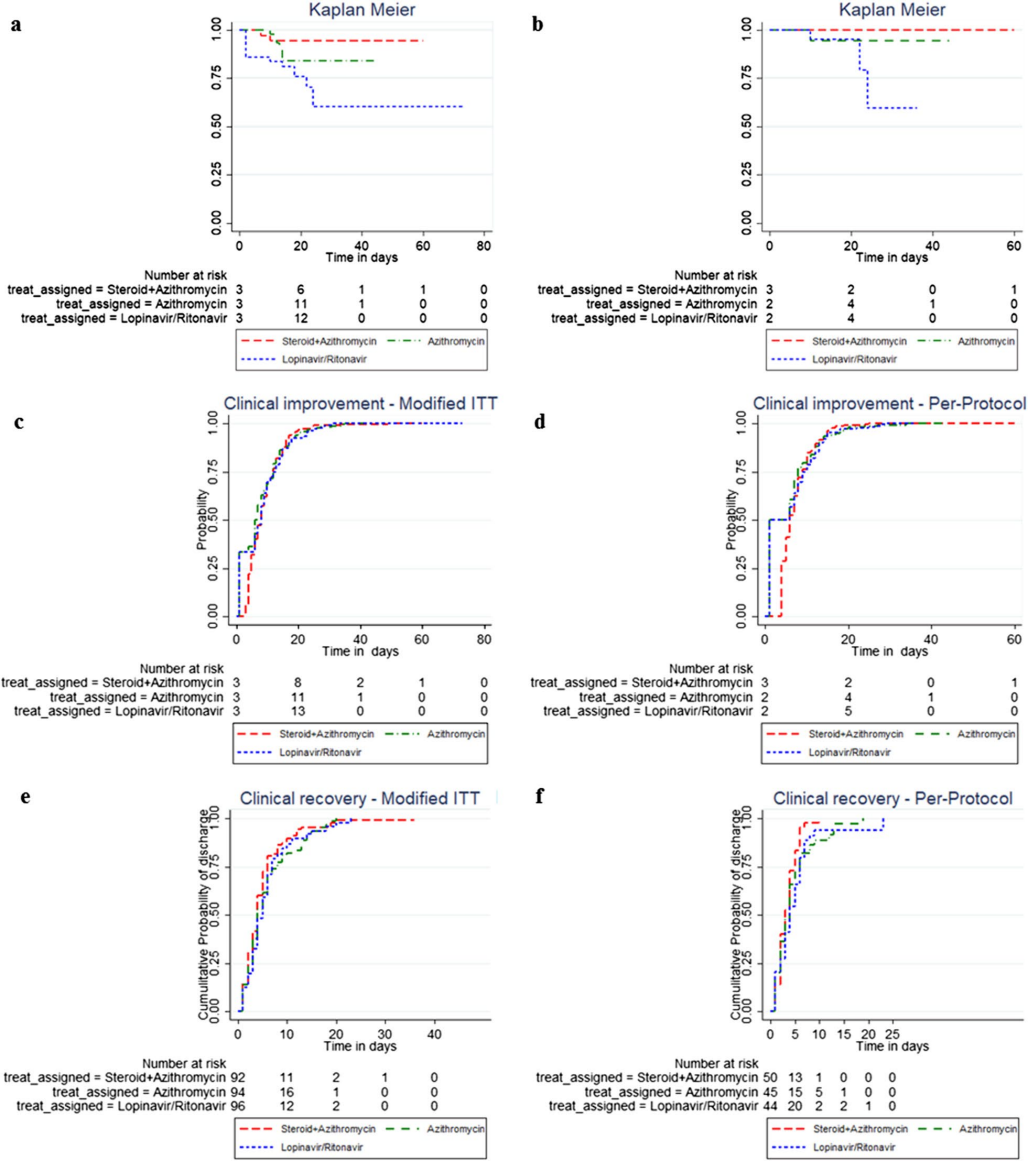
Kaplan–Meier and Nelson-Allen curves for Modified ITT and perprotocol populations by intervention groups; death/mechanical ventilation in the Modified ITT (Kaplan–Meier estimate, part **a**), and perprotocol populations (Part **b**), cumulative probability of discharge in Modified ITT and perprotocol population (Nelson-Allen, parts **c** and **d**), cumulative probability clinical recovery in the Modified ITT population and perprotocol populations (Parts **e** and **f**)

The Kaplan–Meier curves in the Modified ITT population and the per-protocol population are shown in Fig. 2. Log rank test showed no statistically significant difference in time to death/intubation in the two groups of I and II in any of the populations, when compared with Lopinavir/Ritonavir regimen (P = 0.53 and 0.34; Table 7). In the Cox proportional Hazard model, treatment groups I and II independently did not show a statistically significant difference compared with treatment group III in terms of time to death/mechanical ventilation among both populations.

**Table 7:** The log-rank tests for death/mechanical ventilation experience, discharge, time to clinical recovery in the modified ITT and perprotcol populations

Treatment groups	Steroid + Azithromycin	Azithromycin	Lopinavir/Ritonavir	Total	Log-Rank test
Number of Death/Intubation in Modified ITT population, n = 268	2	6	6	14	P = 0.53
Number of Death/Intubation in per-protocol population (first definition), n = 133	0	1	3	4	P = 0.34
Number of Discharges in Modified ITT population, n = 268	85	82	80	247	P = 0.335
Number of Discharges in per-protocol population (first definition), n = 133	45	43	38	126	P = 0.055
Number of Clinical improvment in Modified ITT population, n = 282	89	86	87	262	P = 0.291
Number of Clinical improvment in per-protocol population (first definition), n = 139	48	43	42	133	P = 0.098

Hazard ratio for the time to death/intubation among the patients receiving regimen I versus regimen III was 0.43 in the modified ITT population (95% CI: 0.09–2.18). This ratio could not be calculated for the per-protocol population due to the small number of outcomes. As indicated, the study was stopped for some reasons according to the decision of the scientific committee in the DSMB meeting, before reaching a sufficient sample size. It is worth mentioning that no serious violations of proportional hazard assumption were observed in all models.

Among the Modified ITT population, 288 patients were discharged with complete or partial recovery, and 16 were discharged with personal consent on the first day of inclusion. Individuals who were discharged with personal consent were right-censored in the Cox Model and eventually excluded. However, the distribution of excluded subjects was approximately equal in the two groups (see participants flow). The mean length of stay in hospital among those discharged in the modified ITT population was calculated to be 5.5, 6.4 and 6.4 days in the patients who received regimen I, II, and III, respectively. The mean LOS in the modified ITT population who received prednisolone (regimen I) was significantly lower when compared with group III (p = 0.028). However, there was no statistically significant difference between group II and group III (p = 0.993; Table 7). Furthermore, the differences in the mean LOS values of the per-protocol populations in group I (p = 0.007) and group II (p = 0.595) were not statistically significant when compared with Lopinavir/Ritonavir regimen (group III), (see Table 8).

**Table 8:** Comparison of length of hospital stay in the Modified ITT and perprotocol populations

	Treatment groups	N	Length of stay in days			P-value
			Mean	SD	Range	
Modified ITT	Steroid + Azithromycin	89	5.5	3.1	1–19	0.028
	Azithromycin	90	6.4	4.0	1–28	0.993
	Lopinavir/Ritonavir	89	6.4	3.2	1–20	
per-protocol	Steroid + Azithromycin	48	4.4	1.9	1–10	0.0007
	Azithromycin	43	5.9	4.2	1–28	0.595
	Lopinavir/Ritonavir	41	5.8	2.0	1–10	

The Nelson-Aalen curves (cumulative probability of discharge cases) for the Modified ITT and per-protocol populations are shown in Fig. 2. Also, Log rank test showed no statistically significant differences in the time to discharge of patients in both populations of regimen I and II when compared with regimen III (Log-Rank test: P = 0.335; P = 0.055; Table 7).

In the Cox proportional Hazard model, both populations in treatment groups I and II independently did not show any statistically significant difference when compared with treatment group III in terms of time to discharge on clinical judgment in both populations. Hazard ratios for the time to discharge in regimen I versus regimen III and regimen II versus regimen III group were 0.43 and 1.01, respectively in the modified ITT population (95% CI: 0.09–2.18 and 0.32–3.14) and 1.53 and 1.02, respectively in the per-protocol population (95% CI: 0.98–2.39 and 0.65–1.58). No serious violations of the proportional hazard assumption were observed in all models.

Regarding time to clinical recovery, the Nelson-Aalen curve was plotted for the Modified ITT and per-protocol populations to depict cumulative probability of clinical recovery over the study period (Fig. 2). Based on Log-Rank analysis, no statistically significant differences were observed between the populations in all three groups in terms of clinical recovery declared by the attending physician (P = 0.291; P = 0.098; Table 7).

In the Cox proportional Hazard model, there was also no significant difference in the time to clinical recovery in regimen I and II versus regimen III in both populations (HR = 1.21 and 1.01 [CI: 0.90–1.63 and 0.75–1.36]; HR = 1.47 and 1.10 [CI: 0.96 -2.45 and 0.71–1.69[). There were no serious violations of proportional hazard assumption in any of the models.

Although more adverse drug reactions were found to be linked to Lopinavir/Ritonavir treatment regimen (III) when compared with the other regimens, no statistically significant differences were found in adverse effects in the three groups.

No anaphylactic reactions were observed in regimen II group. Also, no neurologic adverse events were observed in patients who received treatment regimen I (Table 9).

A comparison of total symptom changes and the need for oxygen support during hospitalization using GEE models is summarized in Table 10. As can be seen in Table 10, the 3 groups had almost the same total symptom change (P = 0.283) and need for oxygen support (P = 0.862) during hospitalization.

**Table 9:** Comparison of adverse events by treatment groups in the Modified ITT population

Adverse event	Steroid + Azithromycin N = 105 n (Incidence %)	Azithromycin N = 100 n (Incidence %)	Lopinavir/Ritonavir N = 110 n (Incidence %)	Total N = 315 n (Incidence %)	P-value
Treatment group					
Anaphylaxis	1 (0.95)	0 (0)	2 (1.82)	3 (0.95)	0.399
Erythema	1 (0.95)	0 (0)	1 (0.91)	2 (0.63)	
Itching	1 (0.95)	0 (0)	1 (0.91)	2 (0.63)	
Bronchospasm	0 (0)	0 (0)	1 (0.91)	1 (0.32)	
Nausea	0 (0)	0 (0)	1 (0.91)	1 (0.32)	
Wheezing	0 (0)	0 (0)	1 (0.91)	1 (0.32)	
Vomiting	0 (0)	0 (0)	1 (0.91)	1 (0.32)	
Gastrointestinal	11 (10.48)	7 (7)	15 (13.64)	33 (10.48)	0.292
Anorexia	1 (0.95)	1 (1)	3 (2.73)	5 (1.59)	
Nausea	4 (3.81)	2 (2)	7 (6.36)	13 (4.13)	
Vomiting	0 (0)	1 (1)	3 (2.73)	4 (1.27)	
Diarrhea	3 (2.86)	3 (3)	10 (9.09)	16 (5.08)	
Abdominal pain	3 (2.86)	1 (1)	5 (4.55)	9 (2.86)	
Dry mouth	1 (0.95)	1 (1)	2 (1.82)	4 (1.27)	
Neurologic	3 (2.86)	3 (3)	3 (2.73)	9 (2.86)	0.993
Fatigue	1 (0.95)	0 (0)	0 (0)	1 (0.32)	
Headache	1 (0.95)	1 (1)	3 (2.73)	5 (1.59)	
Unbalanced	0 (0)	1 (1)	0 (0)	1 (0.32)	
Ophtalmic	0 (0)	0 (0)	1 (0.91)	1 (0.32)	0.393
Endocrinologic	0 (0)	0 (0)	0 (0)	0 (0)	N/A
Cardiac	0 (0)	0 (0)	1 (0.91)	1 (0.32)	0.393
Cardiomyopathic	0 (0)	0 (0)	1 (0.91)	1 (0.32)	
Hematologic	2 (1.9)	0 (0)	0 (0)	2 (0.63)	0.134
Leukopenia	1 (0.95)	0 (0)	0 (0)	1 (0.32)	
Anemia	1 (0.95)	0 (0)	0 (0)	1 (0.32)	
Respiratoric	5 (4.76)	3 (3)	2 (1.82)	10 (3.17)	0.466
Dyspnea	3 (2.86)	2 (2)	2 (1.82)	7 (2.22)	
Coughing	4 (3.81)	1 (1)	1 (0.91)	6 (1.9)	
Dermatologic	0 (0)	0 (0)	2 (1.82)	2 (0.63)	0.153
Skin rash	0 (0)	0 (0)	1 (0.91)	1 (0.32)	
Nephrologic	0 (0)	1 (1)	3 (2.73)	4 (1.27)	0.195
Enuresis	0 (0)	1 (1)	0 (0)	1 (0.32)	
Polyuria	0 (0)	1 (1)	1 (0.91)	2 (0.63)

**Table 10:** Comparison of change of signs and need of oxygen support during hospitalization using GEE models

	Group	β per day hospitalization	SE	P-value	
				With in	Between
Total Signs	Steroid + Azithromycin	− 0.363	0.0717	< 0.001	0.283
	Azithromycin	− 0.442	0.0584	< 0.001	
	Lopinavir/Ritonavir	− 0.520	0.0528	< 0.001	
Need of Oxygen support	Steroid + Azithromycin	− 0.073	0.0523	0.162	0.862
	Azithromycin	− 0.107	0.0459	0.020	
	Lopinavir/Ritonavir	− 0.083	0.0435	0.058	

## Discussion

Our findings showed that low-dose prednisolone decrease the LOS among patients suffering from moderate to severe COVID-19.

The results of our study indicate that the number of deaths, percentage of ICU admission and need for mechanical ventilation in the Modified ITT population were not significantly better in the steroid group (I) who received 25 mg prednisolone for 5 day (4, 5 and 2 cases, respectively), when compared with the Lopinavir/Ritonavir group (6, 7 and 4 cases). In the per-protocol population, similar outcomes were found, whereas the differences were not statistically significant.

The recovery trial demonstrated a lower 28-day mortality rate for COVID-19 patients receiving 6 mg of dexamethasone once daily for up to 10 days [14], while no beneficial effect was found in 28-day mortality and other secondary outcomes among patients receiving methylprednisolone 0.5 mg/kg twice daily for 5 days as reported by METACOVID trial [20]. Another multicenter study reported that > 0.5 mg/kg daily of prednisone did not have any significant effect on the mortality rate in patients with COVID-19 [21].

Based on the controversial findings of these trials, it can be hypothesized that higher doses of corticosteroids may lead to a possible harm rather than beneficial to COVID-19 patients [21]. As a matter of fact, low-dose corticosteroids may be potentially associated with a lower mortality rate. It should be taken into consideration that the efficacy of glucocorticoids is dependent on dosing, timing of corticosteroids initiation, the duration of use, underlying medical condition, and disease severity in the right patient.

In the present study, the mean LOS in the modified ITT and per-protocol population was 5.5 and 4.4 days, respectively in group I as compared to6.4 and 5.8 days in the Lopinavir/Ritonavir group (group III). The differences between the two population in both groups were statistically significant (p = 0.028; p = 0.0007). The discharge experience was similar in the three groups among the modified ITT and per-protocol population (Log-Rank test: P = 0.335; P = 0.055). The time to clinical recovery of patient groups was found to be similar in both populations (p = 0.291; p = 0.098). The probability of clinical recovery over the study period was not better in either regimen I or II compared with regimen III in both populations (HR = 1.21 and 1.01 [CI: 0.90–1.63 and0.75–1.36]; HR = 1.47 and 1.10 [CI: 0.9 6 -2.45 and 0.71–1.69]).

Furthermore, treatment with azithromycin alone showed no clinical or statistical differences in any of the studied outcomes compared with Lopinavir/Ritonavir.

The occurrence of adverse events in the Lopinavir/Ritonavir group (III) was higher than in the steroid group (I), and the most common drug complication in this study was gastrointestinal side effects, but no statistically significant differences were found in adverse effects among the patients receiving regimens I or II compared with regimen (III).

In the present study, we designed a trial that could reveal any potential therapeutic benefits of steroids. Patients were recruited to our study if they had been diagnosed with COVID-19 pneumonia. At this stage of the disease, the virus may have completely spread and replicated in the body, leading to inflammatory response. Therefore, least is expected for a proper therapeutic response to antiviral drugs alone in the inflammation-driven damaging phase when patients have already passed viral replication stage of the disease [22, 23]. Anti-inflammatory strategy might be less useful for consolidated and irreversible tissue damage [24], therefore, appropriate therapeutic strategy at the right time is of great importance.

Pathological evidences have revealed pulmonary edema and hyaline membrane formation as well as an early stage of acute fibrinous and organizing pneumonia (AFOP) in critically ill COVID patients [6–8], indicating a specific form of acute lung injury and steroid-sensitive pathology.

Association between cytokine storm and severity and clinical outcomes of patients with COVID-19 has been reported in many studies, suggesting a possible beneficial effects of corticosteroid therapy which can decrease inflammation-induced lung injury [5, 11, 25].

Due to the lack of approved drug for the treatment of COVID-19 patients and the fatality of the disease, it was ethically problematic to prevent physicians from recommending a particular intervention or a specific treatment regimen. Hydroxychloroquine and lopinavir/ritonavir have been previously recommended by Iranian and some other national guidelines during early stages of the pandemic [26, 27]. As an agreement, all clinical units in our study were asked to continue using additional treatment modalities (Table 5) and these modalities were equal in all three intervention groups as decided by the DSMB, and equal treatment change rules were considered regardless of which therapeutic group they were recruited to. It is noteworthy that the study was conducted in an early phase of the COVID-19 pandemic, when the use of hydroxychloroquine, azithromycin and lopinavir/ritonavir was under study.

One of the most challenging issues for physicians in treating patients with acute viral diseases, especially COVID19, is the use of corticosteroids in the inflammatory phase of hospitalized patients. During the course of this study, the release of the results of similar studies published around the world on the efficacy of corticosteroids in controlling the symptoms and inflammatory phase of the disease increased the desire of the physicians to use steroid-based therapies. It even seems that the physicians moved from low-dose corticosteroids to pulse steroid therapy. This led to a violation of the treatment protocol, reduced stay of patients in the ITT population, and ultimately prolonged the course of the sampling in this study.

Of the 336 patients in the Modified ITT population, 18 cases were admitted to the intensive care unit (5%), and a total of 16 patients died during the trial which was equivalent to less than 5% of patients recruited. This rate is much lower than the initial expectations and published national and global data, indicating the inclusion of patients with better general condition in the study. In other words, the physicians and the treatment team did not include patients with more severe and worsening conditions. This shows that multicentral clinical trial studies require more and closer support of the scientific committee in critical circumstances.

## Limitations

This study has several limitations. The study was stopped with a limited sample size according to the decision of the scientific committee in the DSMB meeting, before reaching a sufficient sample size. Due to the pandemic conditions of the disease and compatible clinical findings with the COVID-19 disease, CT scan findings were considered diagnostic by physicians, which is in accordance with the national protocol of COVID-19. Sixteen participants were excluded after randomization because they discharged with personal consent on the first day of inclusion. This study was conducted at multi center-hospitals with additional drugs prescription due to the unavailability of specific treatment and ethical issues, therefore, what was emphasized in this study was that these additional treatments should be indicated on the same condition in all three groups. The use of corticosteroids in oral, intravenous and pulse therapy forms (i.e., additional drugs) in the comparison groups was based on physicians' opinion so as not to deprive patients of appropriate treatment when corticosteroids were recommended. According to the description of the methods, the protocols for treatment duration in the schemes 1 and 2 were of 5 days, which could be continued up to 10 days if necessary. There may also be a potential source of bias for extending the treatment time.

The treatment protocols in these groups were continued for 10 days based on patients' response to treatment (criteria for discharge) if needed. Thus, this was the description of what were the criteria for extending treatment time.

## Conclusion

The present study showed clinical benefits of a therapeutic regimen based on low-dose prednisolone for COVID-19 patients, where the only benefit found was in relation to the length of stay in the hospital in patients with moderate to severe COVID-19 compared to other regimens. The steroid sparing effect may be utilized to increase the effectiveness of corticosteroids in the management of diabetic patients by decreasing the dosage.

## Data Availability

Available upon request.

## Abbreviations

LOS: Length of hospital stay
mITT: Modified intention-to-treat
ICU: Intensive care unit
NSAIDs: Nonsteroidal anti-inflammatory drugs
DSMB: Data and Safety Monitoring Board
AFOP: Acute fibrinous and organizing pneumonia
PH: Proportional hazard,
GEE: General Estimation Equation
